# Prevalence of failed induction of labor and associated factors among women who underwent induction of labor in Ethiopia: A systematic review and meta-analysis

**DOI:** 10.1371/journal.pone.0305384

**Published:** 2024-11-15

**Authors:** Mulat Ayele, Befkad Derese Tilahun, Gizachew Yilak, Abebaw Alamrew, Amare Zewdie, Eyob Shitie Lake

**Affiliations:** 1 Department of Midwifery, College of Medicine and Health Science, Woldia University, Woldia, Ethiopia; 2 Department of Nursing, College of Medicine and Health Science, Woldia University, Woldia, Ethiopia; 3 Department of Public Health, College of Medicine and Health Science, Wolkite University, Wolkite, Ethiopia; Debre Markos University College of Health Science, ETHIOPIA

## Abstract

**Background:**

The occurrence of failed induction led to higher rates of health problems and death among mothers, mainly as a result of complications related to cesarean delivery, such as postpartum bleeding, morbidly adherent placenta and surgical site infection. Even though a systematic review and meta-analysis were done before July 2020 with limited studies, there were varies inconsistent studies after that and no updated summarize evidence about the issue as a nation. Therefore, this systematic review and meta-analysis aimed to assess the current pooled prevalence of failed induction and its associated factors in Ethiopia by including multiple inconsistent studies.

**Methods:**

Comprehensive literature was searched in PubMed, Google Scholar, and HINARI from January 1, 2013, to September 23, 2023. A random effect model was used to estimate pooled prevalence and adjusted odds ratio. Stata (version17.0) was used to analyze the data. Cochrane Q-test and I squared statistics were computed to assess heterogeneity among studies. A sub group analysis was done based on study region to minimize underling heterogeneity. Funnel plot and Eggers test were done to assess publication bias and corrected by trim and fill analysis.

**Result:**

Overall, one thousand fifty-two articles were retrieved and finally twenty-eight studies were included in this systematic review, including 9757 participants. The pooled prevalence of failed induction of labor was 22.39% (95% CI: 21.57–23.21). Subgroup analysis showed that failed induction of labor was highest in Addis Ababa and lowest in Tigray region. Rural residence (AOR = 3.31, 95% CI: 2.39–4.57), nullipara women (AOR = 2.63, 95% CI: 2.14–3.24), unfavorable bishop score (AOR = 3.98, 95% CI: 2.19–7.08), hypertensive disorder during pregnancy (AOR = 3.63, 95% CI: 2.69–5.01) and premature rupture of membranes before the onset of labor (AOR = 2.51, 95% CI: 1.5–4.26) were significantly associated with failed induction of labor.

**Conclusion:**

The pooled prevalence of failed induction of labor in Ethiopia was high. Unfavorable bishop score, nulliparous, rural residence, women who had premature rupture of membrane and hypertensive disorder during pregnancy were significantly associated with failed induction of labor. Therefore, Healthcare providers or obstetricians should consider proper cervical assessment for bishop score before the initiation of induction. The Ministry of Health ought to create a distinct set of guidelines specifically addressing the cervical ripening and/or induction protocol for women who experienced premature rupture of membranes (PROM) and had a hypertensive disorder during pregnancy, especially those who were administered magnesium sulfate (MgSO4).

## Introduction

The process of stimulating contractions before the natural onset of labor, with or without ruptured membranes, is referred to as induction of labor. Its purpose is to imitate the natural labor process by initiating it artificially through cervical ripening and uterine contractions before it occurs naturally [1,2]. Induction of labor is typically recommended by healthcare professionals when a woman reaches 41 completed weeks of gestation or more and has conditions such as pre-labor rupture of amniotic membranes, hypertensive disorders, maternal medical complications, fetal death, fetal growth restriction, chorioamnionitis, multiple pregnancies, vaginal bleeding, or other complications [2,3]. Various techniques are employed for cervical ripening and induction, including the administration of oxytocin, misoprostol, and dinoprostone. Other methods encompass artificial rupture of membranes, membrane stripping, extra-amniotic saline infusion, the use of trans cervical balloons, and hygroscopic cervical dilators [1,2]. However, the success of induction is not guaranteed, and it can result in failure. Failed induction is defined as the inability to achieve regular contractions (e.g., every 3 minutes) and cervical changes after at least 6–8 hours of administering oxytocin, along with the artificial rupture of membranes if possible [1]. The Bishop score is the most commonly used method for predicting induction success. In this scoring system, the clinician evaluates the cervical dilatation, effacement, consistency, and position of the cervix. If the bishop score is below 6 out of 10 scoring system the cervix is unfavorable and if induction is done without cervical ripening the failure rate of induction becomes high [1,4]. Failed induction increases the risk of operative deliveries and complications for both the mother and the fetus such as postpartum hemorrhage, surgical site infection, morbidly adherent placenta for future pregnancy and respiratory difficulty of the newborn [1]. The American College of Obstetricians and Gynecologists also recommend health care providers should be used cervical ripening methods when labor is induced in women with an unfavorable cervix as a safe prevention of the primary cesarean delivery secondary to failed induction [5]. The failure rates of inductions vary across different healthcare settings. In Assam Medical College and Hospital in Dibrugarh, India, the prevalence of failed induction is reported to be 67.2%) [6]. In Ferrara University, Italy, it is 19.4% [7]. In Nigeria, the prevalence is 36.5% [8], while in Tanzania, it is 19% [9]. In Ethiopia, the rates range from 11.8% in the Tigray region to 43.2% in Wolliso St. Luke Catholic Hospital in Addis Ababa [1,10–16]. This increases failed induction related caesarean delivery. for instance the recent study done in north west Amhara region referral hospitals showed that out of 41 induced nulliparous women, 10(58.8%)of women were delivered by caesarean delivery for the indication of failed induction [17]. Factors associated with failed induction of labor include being a first-time mother, having an unfavorable Bishop score, being younger than 24 years old, having a fetal weight greater than 3500 grams, experiencing a non-reassuring fetal heart rate pattern, residing in a rural area, having premature rupture of membranes (PROM), having hypertensive disorders during pregnancy, and gestational age [10–12,15,16].

Although a previous systematic review and meta-analysis was conducted [18], it was not representative on a national level as it included a limited number of studies. Moreover, it did not evaluate the current prevalence of failed induction as it only considered studies conducted before July 2020. Since then, there have been inconsistent studies assessing the proportion and associated factors of failed induction. Therefore, the purpose of this systematic review and meta-analysis is to provide a comprehensive summary of the current pooled prevalence of failed induction and its associated factors in Ethiopia by including multiple studies.

## Methods

### Study design and setting

A systematic review and meta-analysis were conducted to investigate the occurrence of failed induction of labor in Ethiopian mothers who were in the process of giving birth. The study adhered to the Preferred Reporting Items for Systematic Review and Meta-Analysis (PRISMA) Guidelines, which consist of checklists that provide guidance for conducting and reporting systematic reviews and meta-analyses (S1 File). These guidelines aim to enhance transparency and accuracy in reviews conducted across various disciplines, including medicine [19]. Ethiopia, classified as a low-income country, is located in the Horn of Africa and is projected to have a population of 123.4 million in 2022, 133.5 million in 2032, and 171.8 million in 2050. From an administrative perspective, Ethiopia is divided into 11 regions and two city administrations. The regions are further subdivided into zones, and zones are further divided into districts. Finally, districts are divided into kebeles, which represent the smallest administrative divisions and typically have a population ranging from 2000 to 3500 residents.

### Search strategies and sources of information

We have conducted a search in the PROSPERO database (https://www.crd.york.ac.uk/prospero/) to determine if any recently published or ongoing projects exist on the same topic, in order to avoid unnecessary duplication. Our search revealed that there were no ongoing or published articles related to this specific topic. Therefore, we registered this systematic review and meta-analysis in the PROSPERO database with the ID number CRD42023442768. To gather relevant articles, we conducted a comprehensive literature search using international databases such as PubMed, Google Scholar, and HINARI from September 9 to 17, 2023. Additionally, we searched for grey literature using Google. We formulated search terms following the PICO guidelines and utilized different Boolean operators ’AND’ and ’OR’. The search terms used were "prevalence", OR "magnitude", OR "proportion", AND "associated factor", OR "determinant", OR "factors", AND "failed induction", OR "induction of labor", OR "induction", AND "laboring mother", OR "pregnant mother", OR "induced women", AND "Ethiopia"(S2 File).

### Inclusion criteria

This systematic review and meta-analysis focused on articles that met specific criteria. We included studies that reported the prevalence or proportion of failed induction and the associated factors, as well as studies that reported the success of induction. Both gray literature and published articles written in the English language were considered. We specifically looked for observational study designs, such as case-control and cross-sectional studies, that reported the prevalence or proportion of failed induction and its associated factors. The included unpublished literatures were found from repositories of Addis Ababa University, Harmiya University, Debre Brihan University, and University of Gondar through searching from their repositories web site. The timeframe for inclusion was from January 1, 2013, to September 23, 2023.

Although there were previous reviews on the topic from September 20, 2005, to July 29, 2020, they were not comprehensive and up to date. Therefore, we expanded the inclusion timeframe to include studies from January 1, 2013, to September 23, 2023. This decision was made because there have been numerous inconsistent studies conducted during this extended period, providing more recent and relevant evidence for our review.

### Exclusion criteria

In our selection process, we excluded articles that did not have full abstracts or complete texts available. We also excluded systematic reviews, meta-analyses, and articles that did not report on the outcome of interest. These exclusion criteria were applied to ensure that we included only relevant and complete studies in our systematic review and meta-analysis.

### Outcome of measurement

The first outcome of this systematic review and meta-analysis study was focused on failed induction of labor. Failed induction is defined as the inability to achieve regular contractions and cervical change after a minimum of 6–8 hours of receiving the maintenance dose of oxytocin, along with the option of artificial rupture of membranes if feasible [1]. The second outcome of this study aimed to identify the associated factors of failed induction. The goal was to examine the factors that may contribute to the occurrence of failed induction in labor. The systematic review and meta-analysis sought to analyze and summarize the available evidence on these associated factors to provide a comprehensive understanding of their influence on induction outcomes.

### Data extraction

The datasets were exported to Mendeley Reference Manager, and from there, they were transferred to a Microsoft Excel spreadsheet for further analysis. The first step in the analysis process involved removing any duplicate data from the review. To ensure accurate data extraction, three authors (MA, ESL, and AA) independently extracted all the relevant data using a standardized data extraction form which was adapted from the JBI (Joanna Briggs Institute) data extraction format [20]. In cases where there were disagreements between the reviewers, a second team of reviewers (GY, BDT, and AZ) was involved to resolve the discrepancies. The resolution process involved critical discussions and evaluations of the articles by the independent group of reviewers. The following information was extracted from the articles: author names, sample size, publication year, study area, region, study design, prevalence of failed induction, and adjusted odds ratios with a 95% confidence interval for factors associated with failed induction of labor. By following this systematic data extraction process, the study aimed to ensure consistency and accuracy in capturing the relevant information from the selected articles.

### Quality assessment

To assess the quality of the included studies, the Newcastle-Ottawa Quality Assessment Scale (NOS) was used for both cross-sectional and case-control study designs [21] (S3 File). Three authors (MA, BDT, and GY) were responsible for independently assessing the quality of each study. The assessment covered various aspects, including methodological quality, sample selection, sample size, comparability, outcome assessment, and statistical analysis of the study.

In cases where there were disagreements among the three authors during the quality assessment, two additional authors (ESL and AZ) were involved. The disagreements were discussed and resolved through thorough deliberation and consensus among the authors. This process ensured that the quality assessment was conducted in a rigorous and comprehensive manner, considering multiple perspectives and expertise among the author team.

### Data processing and analysis

The extracted data in Microsoft Excel spreadsheet format was imported into STATA version 17 for analysis. A random-effects model was employed to estimate the pooled prevalence of failed induction among laboring mothers in Ethiopia. To assess the heterogeneity among the included studies, I2 statistics were calculated. The I2 value provides an indication of the percentage of variation across studies that can be attributed to heterogeneity rather than chance.

Based on the I2 values, heterogeneity was categorized as follows: 0–40% indicating mild heterogeneity, 40–70% indicating moderate heterogeneity, and 70–100% indicating considerable heterogeneity [22]. Subgroup analysis was conducted based on the study region to explore potential variations in the prevalence of failed induction. To examine the potential risk of publication bias, funnel plots and Egger’s test were performed [23]. The p-value of the Egger’s test (0.0072) indicated the presence of publication bias, as it was less than the significance level of 0.05. Subsequently, trim and fill analyses were conducted to adjust for this bias.

The pooled prevalence and pooled adjusted odds ratios (OR) for factors associated with failed induction of labor among women who underwent induction were presented in a forest plot format. The forest plot included the point estimates of prevalence and OR, along with their corresponding 95% confidence intervals (CI). This format allowed for a visual representation of the pooled results and provided a comprehensive overview of the estimates and their precision.

### Subgroup and sensitivity analyses

Subgroup analyses were performed based on the study region to examine potential variations in the prevalence of failed induction among laboring mothers in different regions of Ethiopia. This analysis aimed to explore whether the prevalence estimates differed significantly across different geographical areas.

In addition, sensitivity analysis was conducted to assess the stability and robustness of the pooled estimates to outliers and the potential impact of individual studies on the overall results. Sensitivity analysis helps evaluate the influence of individual studies on the overall findings by systematically excluding one study at a time and re-analyzing the data. This analysis allows for a better understanding of the potential impact of specific studies on the pooled estimates and the overall conclusions of the systematic review and meta-analysis.

## Result

### Characteristics of the included studies

**Fig 1:**
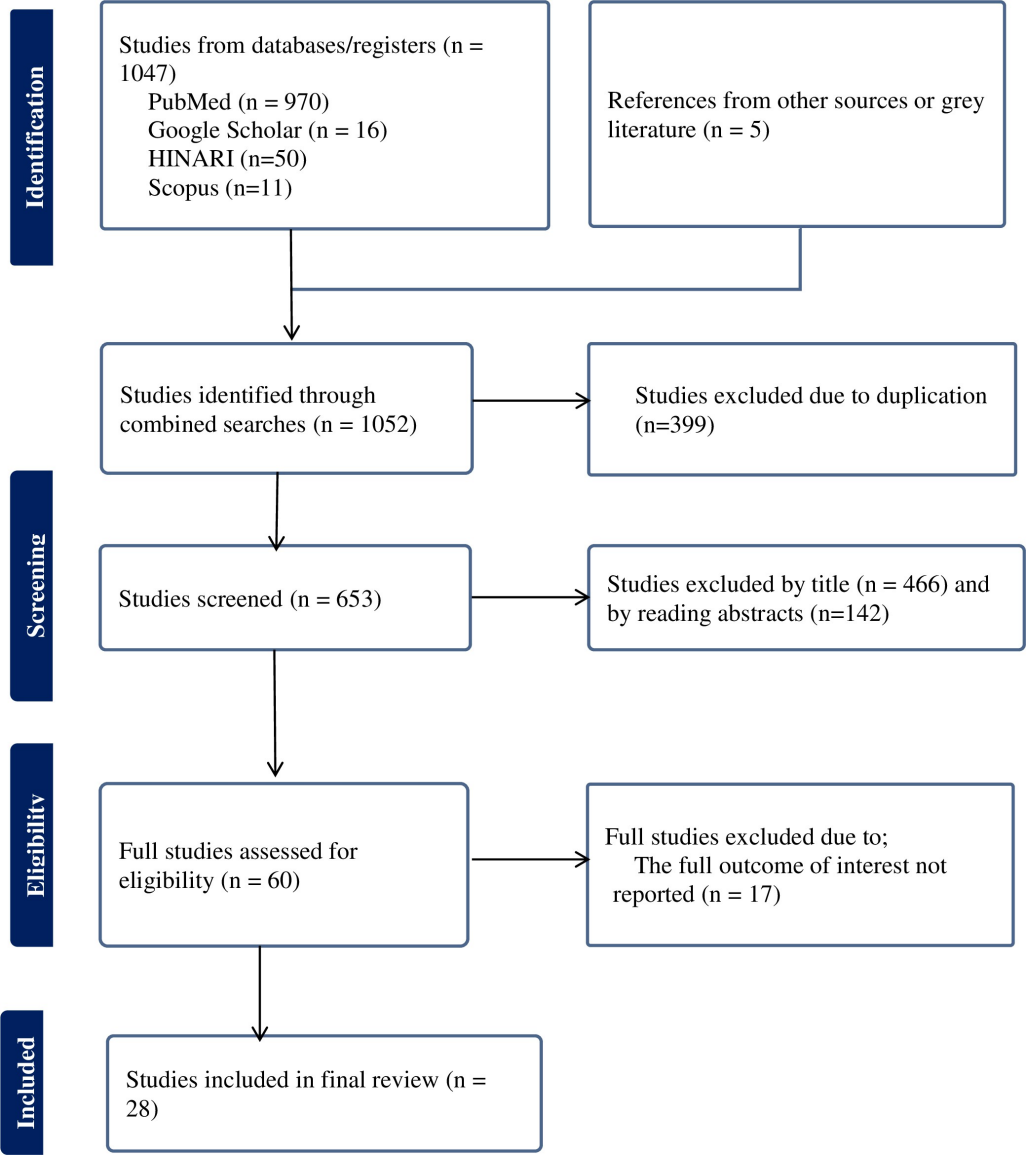
Flow chart of selection for systematic review and meta-analysis on prevalence of failed induction of labor and associated factors among women who underwent induction of labor in Ethiopia.

The search strategy conducted in HINARI, Google Scholar, and PubMed databases yielded a total of 1052 articles since 2013. After removing duplicates, 653 articles remained. Further screening based on titles and abstracts led to the exclusion of 466 and 142 articles, respectively. The remaining articles underwent a full-text evaluation for inclusion criteria, resulting in the exclusion of an additional 17 papers. Ultimately, 28 papers met the eligibility criteria and were included in the final systematic review and meta-analysis (Fig 1).

The included studies were distributed across different regions of Ethiopia, with eight studies conducted in Amhara [12,24–30], seven in Oromia [13,31–36], five in the South Nation Nationality and People Region (SNNPR) [10,37–40], and two each in Addis Ababa [14,41], Harari [11,42], and Tigray [15,43], one each in Somali [44], and Dire-Dawa [45]. Among the included studies, 26 were cross-sectional and two were case-control studies. These studies involved a total of 9757 participants, with varying sample sizes ranging from 60 to 743 participants per study. The prevalence of failed induction among induced mothers ranged from 7.2% to 42.2%.

Regarding the quality assessment of the included studies, all studies scored between 8 and 9 on the Newcastle-Ottawa Quality Assessment scale, indicating good quality (Table 1). This assessment helped ensure that the included studies met rigorous methodological standards and provided reliable data for the systematic review and meta-analysis.

**Table 1:** Characteristics of included studies in the systematic review and meta-analysis on failed induction of labor among women underwent induction of labor in Ethiopia,2023.

S.No	Author	Year	Region	Study design	Sample size	Prevalence of failed induction (%)	Quality
1	T. D. Mulualem and K. T. Assefa (2020) [25].	2020	Amhara	cross-sectional	319	19.70	Good
2	B. T. Debelo et al.	2022	Oromia	cross-sectional	293	20.50	Good
3	T. Z. Debele et al.	2021	Amhara	cross-sectional	484	31.40	Good
4	A. Shiferaw et al.	2021	Dire Dawa	cross-sectional	444	16.30	Good
5	M. A. Assemie et al.	2023	Amhara	cross-sectional	411	29.70	Good
6	Y. M. Beshir et al.	2021	Harerie	cross-sectional	717	35.00	Good
7	Y. Abdulkadiret al.	2017	Oromia	cross-sectional	76	42.20	Good
8	G. D. Lueth et al.	2020	Tigray	cross-sectional	346	7.20	Good
9	A. G. Ejigu and S. H. Lambyo	2021	SNNPR	cross-sectional	441	21.00	Good
10	T. Yosef and D. Getachew	2021	SNNPR	cross-sectional	60	23.30	Good
11	T. H. Abraha et al.	2020	Tigray	cross-sectional	380	11.80	Good
12	T.Tadesse et al.	2022	Amhara	cross-sectional	743	24.40	Good
13	E. T. Bekru and B. E. Yirdaw	2018	SSNPR	cross-sectional	347	39.30	Good
14	M. Mohammed *et al*.	2022	SSNPR	cross-sectional	284	22.20	Good
15	R. D. Gebreyohannes and E. Mesfin	2020	Adis Ababa	cross-sectional	339	25.40	Good
16	M. Desta and A. Duguma	2021	Oromia	cross-sectional	268	21.30	Good
17	E. D. Melkie Abenezer et al.	2019	Amhara	case control	336		Good
18	L. LIDET.	2020	Amhara	cross-sectional	405	34.10	Good
19	A. Mekonnen	2021	Harerie	cross-sectional	308	15.30	Good
20	S. Hiluf	2015	Adis Ababa	cross-sectional	347	41.30	Good
21	W. Girma et al.	2016	Oromia	cross-sectional	280	21.40	Good
22	A. Mebratu *et al*.	2022	Somali	cross-sectional	364	36.80	Good
23	E. A. Demssie	2022	Oromia	cross-sectional	379	29.60	Good
24	B. F. Hurissa et al.	2015	SNNPR	cross-sectional	294	17.30	Good
25	K. A. Kitaba et al.	2022	Oromia	cross-sectional	243	16.90	Good
26	N. Melamed, et al.	2022	Oromia	case control	347	26.20	Good
27	M. Wodaje	2018	Amhara	cross-sectional	89	37.40	Good
28	N. Zahid et al.	2021	Amhara	cross-sectional	413	21.30	Good

#### Prevalence of failed induction of labor in Ethiopia

**Fig 2:**
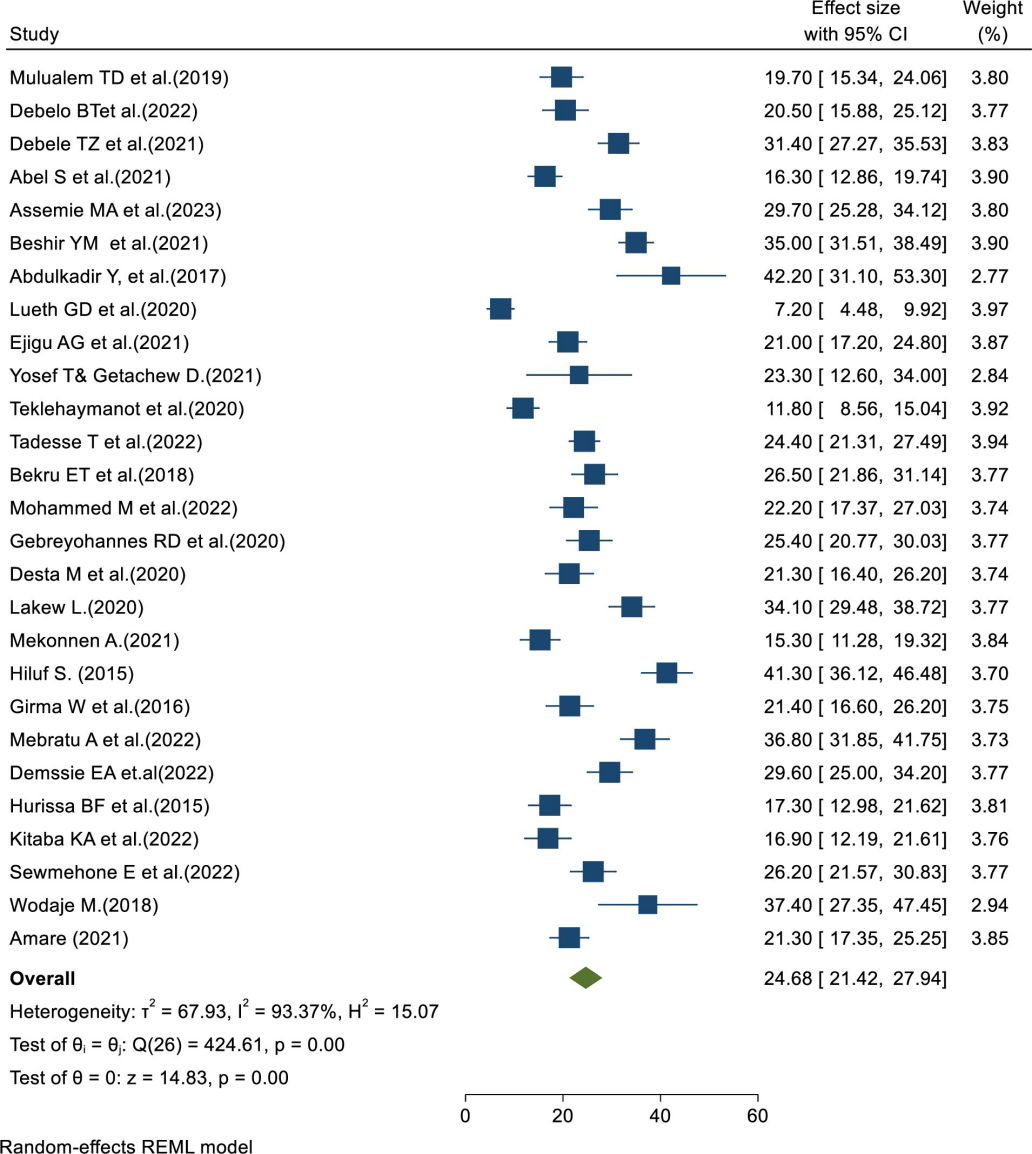
Pooled prevalence of failed induction of labor among women who underwent induction of labor in Ethiopia.

The pooled prevalence of failed induction among women who underwent induction of labor was 24.68% (CI: 21.42–27.94), with the Cochrane heterogeneity index (I^2^ = 93.37%, P = 0.000) showing substantial heterogeneity of different studies (I2> 70%). Therefore, we have used the random effect model to resolve the issue of heterogeneity among the included studies. Additionally, we have considered subgroup analysis as a potential way of addressing heterogeneity. The finding was presented using a forest plot (Fig 2).

#### Sub group analysis of failed induction of labor among mothers who underwent induction of labor in Ethiopia

**Fig 3:**
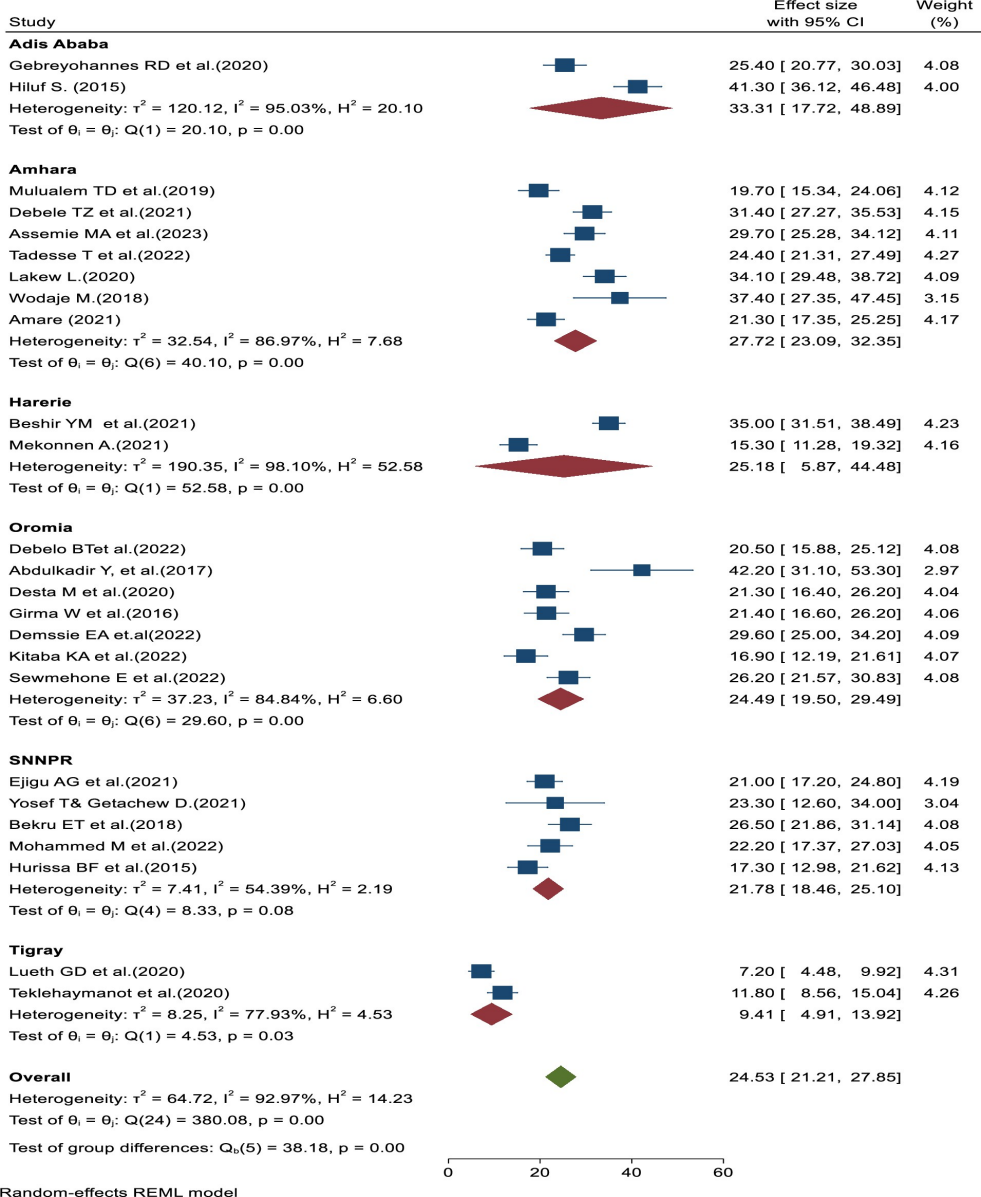
Forest plot showing subgroup analysis of prevalence of failed induction of labor and associated factors among women who underwent induction of labor in Ethiopia.

In this systematic review and meta-analysis, the finding of subgroup analysis by region showed that the pooled prevalence of failed induction of labor among women who underwent induction of labor was highest in Addis Ababa (33.31%; 95% CI: (17.72–48.89), I^2^ = 95.03%, p = 0.00), followed by Amhara region (27.72%; 95% CI: 23.09–32.35), I^2^ = 86.97%, P, = 0.00. However, the sub group analysis result was lowest in Tigray region (9.41%; 95% CI: 4.91–13.92), I^2^ = 77.93%, P = 0.00 (Fig 3). These findings suggest regional variations in the prevalence of failed induction of labor in Ethiopia, with higher rates observed in the Addis Ababa, and Amhara regions, while the Tigray region had the lowest prevalence. The differences may reflect variations in healthcare practices, resource availability, or other regional factors that influence the induction process and its outcomes.

#### Publication bias

**Fig 4:**
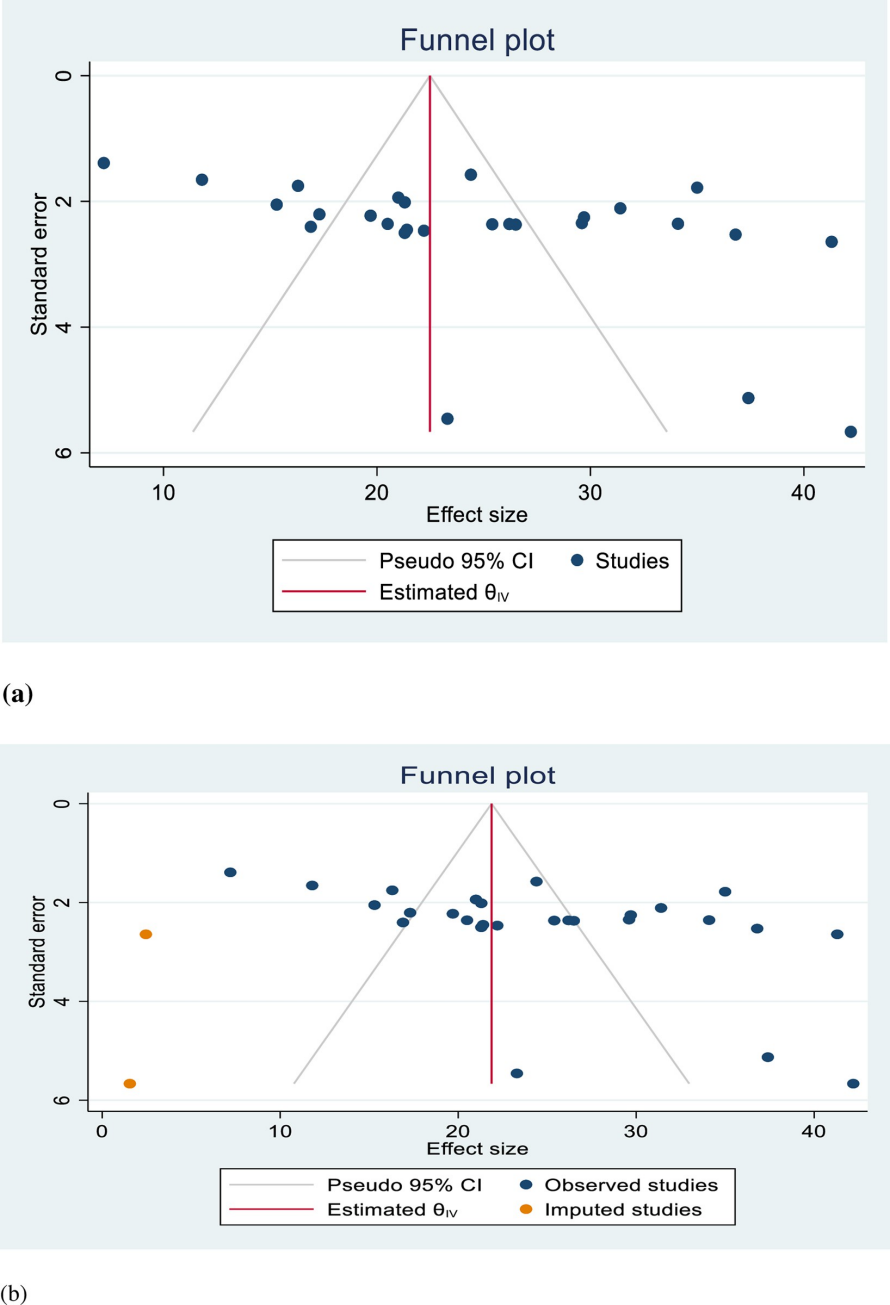
a: Funnel plot for assessing publication bias of the prevalence of failed induction of labor in Ethiopia. b: Funnel plot of the 30 studies’ fill and trim analysis results for adjusting publication bias on prevalence of failed induction of labor in Ethiopia.

In this systematic review and meta-analysis, the presence of publication bias was assessed using a funnel plot, which visually inspected the asymmetry of the distribution of failed induction studies (Fig 4A).

Additionally, Egger’s regression test was conducted, resulting in a p-value of 0.0072 (p < 0.05), indicating the presence of publication bias. To address the publication bias, a non-parametric trim-and-fill analysis was performed among the studies addressing failed induction of labor. This analysis aims to estimate the potential missing studies by trimming the asymmetrical studies from the funnel plot and then filling them symmetrically. After the trim-and-fill analysis, two missed studies were filled in the funnel plot.

After incorporating the imputed missing studies, the total number of studies included in the analysis increased to 30. The computed pooled prevalence of failed induction, based on the imputed studies, was estimated to be 22.39% with a 95% confidence interval (CI) of 21.57% to 23.21%. By conducting the trim and fill analysis, potential publication bias was addressed and accounted for in the estimation of the pooled prevalence of failed induction among women who underwent induction of labor. This analysis provides a more robust and unbiased estimation of the prevalence by imputing the potential missing studies (Fig 4B).

### Sensitivity analysis (Leave-one-out meta-analysis)

**Fig 5:**
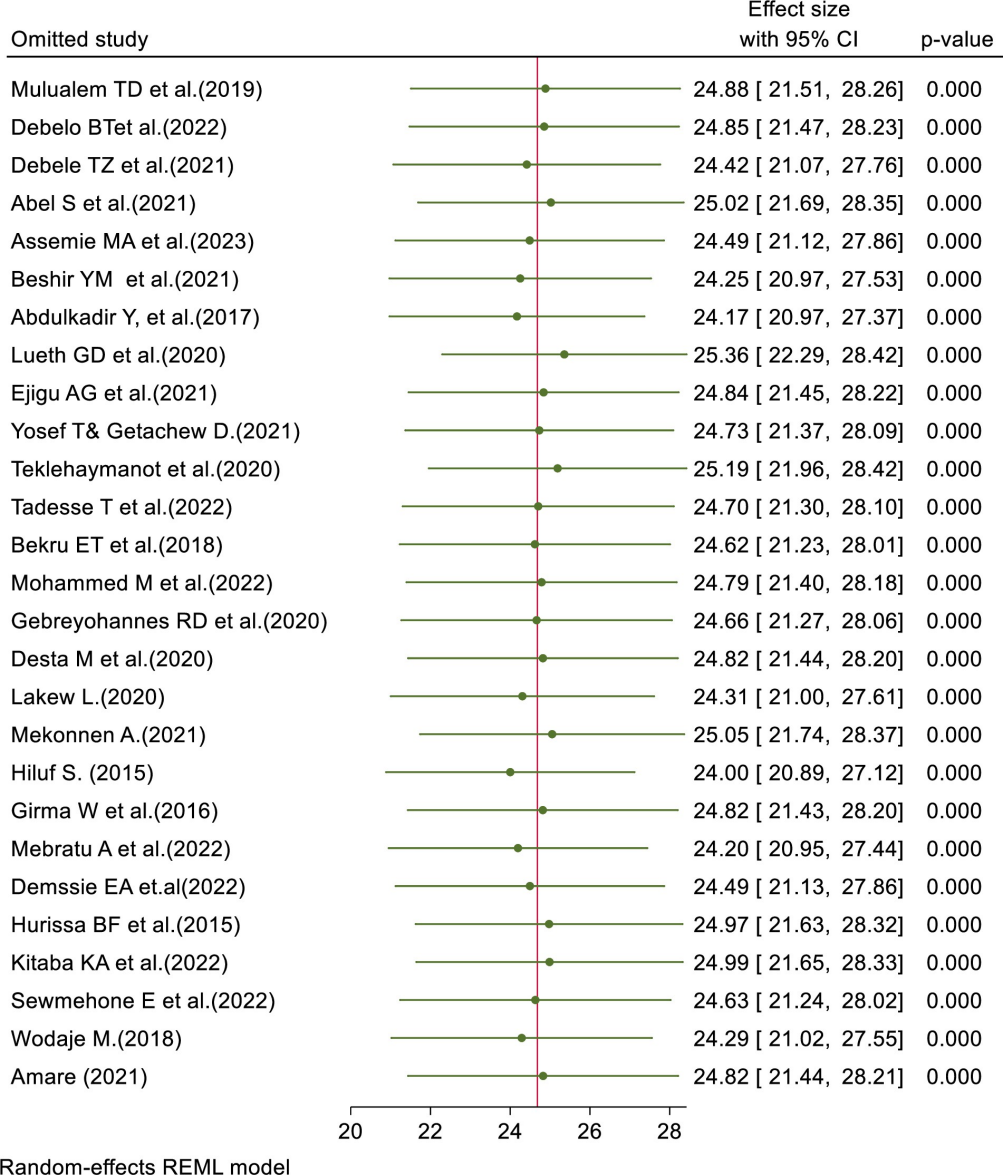
Sensitivity analysis of prevalence of failed induction of labor and associated factors among women who underwent induction of labor in Ethiopia.

The results of the random-effects model indicated that the overall pooled prevalence of failed induction among women who underwent labor induction in Ethiopia was influenced by a specific individual study (Fig 5).

### Determinants of failed induction among women who underwent induction of labor in Ethiopia

In our systematic review and meta-analysis, several factors were found to be significantly associated with failed induction of labor among women in Ethiopia. These factors include residence, parity, bishop score, hypertensive disorder, and premature rupture of membranes (PROM) during pregnancy.

Residing in rural areas was associated with a higher likelihood of experiencing failed induction of labor compared to residing in urban areas. Specifically, women residing in rural areas had 3.31 times higher odds of failed induction (AOR = 3.31, 95% CI: 2.39–4.57).

Nulliparous women, meaning those who had not previously given birth, had a higher risk of failed induction compared to women who had given birth before. Nulliparous women were 2.63 times more likely to experience failed induction (AOR = 2.63, 95% CI: 2.14–3.24).

The likelihood of failed induction was also influenced by the bishop score, which assesses the cervix’s readiness for labor. Women with an unfavorable bishop score had 3.98 times higher odds of failed induction compared to those with a favorable bishop score (AOR = 3.98, 95% CI: 2.19–7.08).

Having a hypertensive disorder during pregnancy was associated with an increased risk of failed induction. Women with hypertensive disorders had 3.63 times higher odds of experiencing failed induction compared to those without hypertensive disorders (AOR = 3.63, 95% CI: 2.69–5.01).

Furthermore, women who experienced premature rupture of membranes before the onset of labor had 2.51 times higher odds of failed induction compared to women without PROM (AOR = 2.51, 95% CI: 1.5–4.26) (Table 2).

**Table 2:** Factors associated with failed induction among women underwent induction of labor in Ethiopia.

Variable	Authors	AOR	95%CI	Pooled AOR	95% CI of pooled AOR
Rural area	T. D. Mulualem and K. T. Assefa (2020) [25] T.Tadesse et al. (2022) [28] M. Mohammed et al. (2022) [39] M. Desta and A.Duguma(2021) [33] A.Mebratu et al. (2022) [45] E. A. Demssie et.al (2022) [34]	4.7 3.7 5.7 2.81 2.28 2.21	1.36–12.8 2.4–5.8 3.12–11.02 1.13–6.99 1.29–4.01 0.94–4.64	3.31	2.39–4.57
Nullipara	T. D. Mulualem and K. T. Assefa (2020) [25] B.T. Debelo et al. (2022) [32] T.Z. Debele et al. (2021) [26] A. G. Ejigu and S. H. Lambyo (2021) [37] T. H. Abraha et al. (2020) [15] T.Tadesse et al. (2022) [28] M. Mohammed et al. (2022) [39] R. D. Gebreyohannes and E. Mesfin (2020) [41] M. Desta and A.Duguma(2021) [33] E. D. Melkie Abenezer et al. (2019) [24] W. Girma et al. (2016) [13] A. Mebratu et al. (2022) [44] E.A. Demssie et.al (2022) [34] B.F. Hurissa et al. (2015) [40] M. Wodaje (2018) [30]	1.72 2.33 1.9 2.35 4.31 2.1 8.4 2.77 2.56 6.24 2.29 2.76 2.32 3.11 4.11	1.67–442 1.26–429 1.2–2.9 1.35–4.09 1.22–15.18 1.2–3.7 2.72–22.36 1.42–4.26 1.58–6.8 3.32–11.73 1.11–4.76 1.55–4.91 1.08–5.02 1.01–9.62 1.3112.86	2.63	2.14–3.24
Unfavorable bishop score	T. D. Mulualem and K. T. Assefa (2020) [25] B.T. Debelo et al. (2022) [32] T.Z. Debele et al. (2021) [26] A. G. Ejigu and S. H. Lambyo (2021) [37] Yosef T& Getachew D. (2021) [38] T. H. Abraha et al. (2020) [15] T. Tadesse T et al. (2022) [28] M. Mohammed et al. (2022) [39] R. D. Gebreyohannes and E. Mesfin (2020) [41] M. Desta and A.Duguma(2021) [33] E. D. Melkie Abenezer et al. (2019) [24] W. Girma et al. (2016) [13] A. Mebratu et al. (2022) [44] E. A. Demssie et.al (2022) [34] B. F. Hurissa et al. (2015) [40] K. A. Kitaba et al. (2022) [35] M. Wodaje M. (2018) [30]	0.147 4.05 2.1 2.37 1.85 10.67 3.4 5.9 66 5.12 11.77 5.28 1.95 3.46 4.54 7.57 3.48	0.06–0.33 1.19–13.77 1.3–3.6 1.16–4.84 1.32–487 1.31–86.87 2.2–5.4 4.52–16.12 5.69–16.4 3.06–7.39 5.19–26.71 2.01–13.88 1.15–3.32 1.51–7.94 1.56–13.19 2.88–19.9 1.15–10.55	3.98	2.19–7.08
HTN disorder during pregnancy	T. Z. Debele et al. (2021) [26] T. Tadesse et al. (2022) [28] R. D. Gebreyohannes and E. Mesfin (2020) [41] A. Mebratu et al. (2022) [44] E. A. Demssie et al. (2022) [34]	2.4 4 8.6 5.11 3.01	1.5–5.1 2.3–7.1 2.39–30.9 2.67–9.79 1.61–5.58	3.63	2.69–5.01
PROM	T. Z. Debele et al. (2021) [26] A. G. Ejigu et al. (2021) [37] T. Tadesse et al. (2022) [28] R. D. Gebreyohannes and E. Mesfin (2020) [41] A. Mebratu et al. (2022) [44] E. A. Demssie et al. (2022) [34] B. F. Hurissa et al. (2015) [40]	0.9 1.06 2.7 4.16 4.88 2.6 5.66	0.4–2 0.54–2.08 1.5–4.6 2.41–9.55 2.33–10.2 1.14–5.91 1.96–16.32	2.51	1.5–4.26

Where; AOR: Is the odds ratio of the respective variable in each primary study.

Pooled AOR: Is the point value of odds ratio when we pooled the AOR of primary studies by our analysis.

95%CI of pooled AOR: Is the 95%CI of the point value of pooled AOR that is the output of our analysis.

## Discussion

In this systematic review and meta-analysis, the pooled prevalence of failed induction of labor among women who underwent induction in Ethiopia was estimated to be 22.39% (95% CI: 21.57–23.21). This finding aligns with a study conducted in Israel, which reported a prevalence of 21.6% [46]. However, our review finding was lower than a study conducted in eight Latin American countries, where the prevalence of failed induction was reported as 30% [47]. Similarly, studies conducted in poor health resource settings reported a prevalence of 24.1% [48], and a study at Kathmandu Medical College reported a prevalence of 34.6% [49], all of which were higher than our findings. These differences could be attributed to various factors such as variations in sample sizes, study locations, induction protocols, and the definition of failed induction used in each study. On the other hand, our review finding was higher than a study conducted in Pakistan, which reported a prevalence of 18% [50]. The disparity between this study and ours might be due to differences in induction and cervical ripening protocols. Pakistan utilized both balloon catheters and PGE2 for cervical ripening, followed by induction with an amniotomy and an oxytocin infusion, which potentially decreased the failure rate of induction compared to our study [50]. In our study, either a balloon catheter or misoprostol was used for cervical ripening, followed by an oxytocin infusion alone for induction. These variations in induction protocols and cervical ripening methods likely contributed to the differences in the prevalence of failed induction observed between our study and the study conducted in Pakistan. Therefore, it is recommended that obstetricians consider utilizing prostaglandin E (PGE) analogues in conjunction with overnight balloon catheters for cervical ripening, followed by induction using oxytocin and amniotomy. This approach is particularly beneficial for promoting labor progression, as PGE analogues have proven effectiveness in both cervical ripening and labor induction. The present review also identified factors that showed a significant association with failed induction of labor in women who underwent induction. Specifically, women with an unfavorable bishop score had approximately four times higher odds of experiencing failed induction compared to those with a favorable bishop score. This finding is consistent with previous studies conducted in Ethiopia [18], Pakistan[50], Avicenna Medical College and Hospital, Lahore [51], Israel [46], and Spain [52], which also reported a higher failure rate of induction of labor among women with an unfavorable bishop score. These findings suggest that assessing the status of the cervix using the bishop scoring system is important. If induction is warranted and the cervix is deemed unfavorable (bishop score less than 6), cervical ripening agents may be used prior to initiating induction in order to reduce the failure rate of induction [1]. Inducing labor in women with an unfavorable cervix has been linked to increased rates of cesarean sections and higher failure rates, especially in nulliparous women. According to recommendations from American College of Obstetrics and Gynecology (ACOG), and Society of Obstetricians and Gynecologists of Canada (SOGC), a cervix is considered favorable if it has a score of 7 or higher, while Federation of Obstetric and Gynecological Societies of India (FOGSI) considers a score of 6 or higher as favorable. When the score is above 8, the likelihood of achieving a vaginal delivery after induction is similar to that of spontaneous labor, as stated in National Institute for Health and Care Excellence (NICE guidelines) [2,53,54]. Thus, obstetricians should give attention for the assessment of bishop score or favorability of cervix before proceeds to induction.

In the present review, it was found that nulliparous women had 2.63 times higher odds of experiencing failed induction compared to multiparous women. This finding is consistent with studies conducted in Pakistan [50], London [55], Israel [46], and Christiana Care Hospital, Newark, DE [56]. This disparity may be attributed to the fact that nulliparous women’s uteruses are less responsive to uterotonics, resulting in poorer uterine activity, which increases the risk of induction failure. Additionally, nulliparous women differ from multiparous women in terms of pre-induction cervical effacement and their response to cervical ripening methods [2,56]. Therefore, obstetricians should take into account that the dosage of cervical ripening methods for nulliparous women should be distinct from that used for multiparous women, just as the induction protocol may require different considerations.

Regarding residence, it was observed that women residing in rural areas had 3.31 times higher odds of experiencing failed induction of labor compared to their counterparts. This is in agreement with the study done in Northern-Tanzania [9]. This could be attributed to the fact that women living in rural areas may have limited access to healthcare facilities, leading to a lack of appropriate and timely interventions. As a result, the majority of inductions given to them are primarily emergency inductions. Due to the urgency of these inductions, the assessment of prerequisites for induction is not adequately conducted. As a result, the failure rate of inductions is higher. Mothers who underwent induction of labor due to hypertensive disorder during pregnancy had 3.63 times higher odds of experiencing failed induction. This finding is consistent with a study conducted at the University of Washington Medical Center [57]. The increased risk of failed induction in this group could potentially be attributed to the administration of magnesium sulfate (MgSo4), a tocolytic drug used for managing conditions like pre-eclampsia or eclampsia. MgSo4 is known to inhibit uterine contractility, which can lead to inadequate progress during labor and ultimately result in failed induction [2]. Moreover, in the case of women with preeclampsia and eclampsia, it is necessary to perform emergency induction within 24 hours and 12 hours respectively. This urgency in induction has an impact on the duration of the process, which is a crucial factor in determining the success or failure of induction [2]. Therefore, it is advisable to opt for elective induction for women diagnosed with hypertensive disorders, particularly preeclampsia, who are admitted to high-risk wards.

Moreover, women who underwent induction of labor due to premature rupture of membranes (PROM) had 2.5 times higher odds of experiencing failed induction. This finding is consistent with a study conducted in Pakistan [50]. The increased likelihood of failed induction in this group can be attributed to several factors. Firstly, PROM can impact the timing for cervical ripening or labor induction, as healthcare providers may be hesitant to allow sufficient time for cervical ripening or reaching the active phase of labor due to concerns about infection. Giving a sufficient time for women who undergone induction of labor decreases the rate of failed induction. ACOG recommended that caesarean delivery for failed induction of labor in latent first stage of labor can be avoided by allowing longer duration of the latent first stage (up to 24 hours or more) and requiring that oxytocin be administered for at least 12–18 hours after membrane ruptured before deciding failed induction of labor [5]. Thus, obstetricians should allow a sufficient time for women who were in induction for the indication of PROM, through giving prophylactic antibiotics for the infection unless intra amniotic infection is diagnosed. Moreover, the time to diagnosis failed induction of labor should be revised. Additionally, there may be reluctance to use mechanical methods to ripen the cervix for fear of infection, which could further contribute to the higher rate of failed induction in women with PROM.

### Implication of the study

This study provides updated national estimates of failed labor induction in Ethiopia. It also offers valuable insights for clinicians and the Ministry of Health, suggesting the use of a combination of cervical ripening and/or induction methods instead of relying on a single method alone to reduce the failure rate. Furthermore, the study indicates the need for revising the assessment criteria for the Bishop score, particularly regarding the definition of an unfavorable score. Increasing the cut-off point for an unfavorable score could potentially improve the success rate of inductions. Lastly, this systematic review and meta-analysis have implications for developing specific cervical ripening and/or induction protocols tailored to mothers who experienced PROM, preeclampsia, and are nulliparous.

### Strength and limitations

One of the strengths of this review is that it incorporates both published and unpublished studies, which helps mitigate the risk of publication bias. Additionally, a significant number of studies from various regions were included, enhancing the representativeness of the findings.

Despite the efforts made to minimize or address potential limitations, it is important to interpret the results in light of certain limitations. Firstly, there were variations in how the outcome of failed induction was measured across the primary studies included in the review, which could introduce some confusion. However, we attempted to accommodate the different outcome definitions used in the included studies within our review. Secondly, the lack of similar reviews conducted in other countries makes it challenging to directly compare our findings with those of other studies, necessitating comparisons primarily with individual primary studies.

## Conclusion

The prevalence of failed induction of labor in Ethiopia was found to be high. Several factors, including an unfavorable bishop score, nulliparity, rural residence, premature rupture of membranes (PROM), and hypertensive disorder during pregnancy, were significantly associated with failed induction of labor. Based on these findings, it is recommended for obstetricians and clinicians to prioritize the assessment of cervix favorability using the bishop score and consider the use of cervical ripening agents for cases with an unfavorable cervix before initiating induction. This approach can potentially help reduce the incidence of failed inductions and improve labor outcomes. Healthcare providers or obstetricians should consider utilizing a combination of prostaglandins and balloon catheters for pre-induction cervical ripening and/or labor induction, instead of relying solely on oxytocin or balloon catheter alone. This combination approach has been shown to reduce the time interval between induction and delivery and decrease the failure rate. The Ministry of Health ought to create a distinct set of guidelines specifically addressing the cervical ripening and/or induction protocol for women who experienced premature rupture of membranes (PROM) and had a hypertensive disorder during pregnancy, especially those who were administered magnesium sulfate (MgSO4). Obstetricians should allow a sufficient time for women who were in induction for the indication of PROM, through giving prophylactic antibiotics for the infection unless intra amniotic infection is diagnosed. Moreover, the time to diagnosis failed induction of labor should be revised. Follow up study is needed to assess the effectiveness of our induction protocol compared to other protocols.

## Supporting information

S1 File PRISMA 2020 checklist. (PDF)

S2 File Search strategy for failed induction of labor and associated factors among women who underwent induction of labor in Ethiopia. (PDF)

S3 File Quality assessment Scale for observational studies to assess failed induction of labor and its associated factor in Ethiopia. (PDF)

## Abbreviations

PRISMA: Preferred Reporting Items for Systematic Review and Meta-Analysis
PROM: Premature Rupture of Membrane
HTN: Hypertension
SNNPR: South Nation Nationality and People Region
