# Supplementary material for: Prevalence of failed induction of labor and associated factors among women who underwent induction of labor in Ethiopia: A systematic review and meta-analysis
Source: PLoS One. 2024 Nov 15;19(11):e0305384. doi: 10.1371/journal.pone.0305384 (PMC11567538; doi:10.1371/journal.pone.0305384)
Supplement: S2 File — (PDF) [file pone.0305384.s002.pdf]

Supplementary file 2: A searching strategy for failed induction of labor and associated factors among women who underwent induction of labor in Ethiopia.

Databases	Searching terms	Number of studies	Searching date
PubMed	((((((((((((prevalence) OR (magnitude)) OR (proportion)) AND (associated factor)) OR (determinant)) OR (factors)) AND (failed induction)) OR (induction of labor)) OR (induction)) AND (pregnant mother)) OR (laboring mother)) OR (induced women)) AND (Ethiopia).	970	from September 9 to 17, 2023
Google Scholar	"induction of labor in Ethiopia"	16	
HINARI	"induction of labor in Ethiopia"	50	
Scopus	"induction of labor in Ethiopia"	11	
Others databases		5	
Total retrieved		1052	
Included		28	
