# Supplementary material for: Prevalence of failed induction of labor and associated factors among women who underwent induction of labor in Ethiopia: A systematic review and meta-analysis
Source: PLoS One. 2024 Nov 15;19(11):e0305384. doi: 10.1371/journal.pone.0305384 (PMC11567538; doi:10.1371/journal.pone.0305384)
Supplement: S3 File — (PDF) [file pone.0305384.s003.pdf]

Supplementary table 3: Newcastle-Ottawa Quality Assessment Scale for observational studies to assess failed induction of labor and its associated factor in Ethiopia

Tble1: Newcastle-Ottawa Quality Assessment Scale for cross sectional studies used in the systematic review and meta-analysis 2023

	Selection				Comparability	Outcome		Total score
Authors	Represe ntativen ess s (1)	Sample size (1)	Non-responde nts (1)	Ascertain ment of the exposure (risk factor) (2)	The subjects in different outcome groups are comparable, based on the study design or analysis. confounding factors are controlled (1)	Assessm ent of the outcom e (2)	Statistic al test (1)	
Mulualem TD et al.(27)	1	1	1	2	1	2	1	9
Debelo BT et al.(28)	1	1	1	2	1	2	1	9
Debele TZ et al.(29)	1	1	1	2	1	2	1	9

Abel S et al.(30)	1	1	1	2	1	2	1	9
Assemie MA et al.(31)	1	1	1	2	1	2	1	9
Beshir YM et al.(5)	1	1	1	2	1	2	1	9
Abdulkadir, Y et al.(32)	1	0	1	2	1	2	1	8
Lueth GD et al.(33)	0	1	1	2	1	2	1	8
Ejigu AG, et al.(34)	1	1	1	2	1	2	1	9
Yosef T, Getachew D et al.(35)	1	0	1	2	1	2	1	8
Tadesse T et al.(36)	1	1	1	2	1	2	1	9
Bekru ET et al.(4)	1	1	1	2	1	2	1	9
Mohammed M et al.(37)	1	1	1	2	1	2	1	9
Gebreyohannes RD et al.(38)	1	1	1	0	0	2	1	7
Desta M et al.(39)	1	1	1	2	1	2	1	9

Lakew L.(40)	1	1	1	2	1	2	1	9
Mekonnen A.(41)	1	1	1	1	1	2	1	8
Hiluf S.(8)	1	1	1	1	1	2	1	8
Girma W et al.(7)	1	1	0	2	1	2	1	8
Mebratu A et al.(42)	1	1	1	2	1	2	1	9
Demssie EA et al.(43)	1	1	1	2	1	2	1	9
Hurissa BF et al.(44)	1	1	1	2	1	2	1	9
Kitaba KA et al.(45)	1	1	1	2	1	2	1	9
Wodaje M (46)	1	0	1	2	1	2	1	8
Amare								

- The scoring process was made according to Newcastle-Ottawa Quality Assessment Scale adapted for cross sectional studies

Selection: (Maximum 5 scores)

1) Representativeness of the cases:

a) Truly representative of the HCC patients (consecutive or random sampling of cases). 1 score

b) Somewhat representative of the average in the HCC patients (non-random sampling) . 1 score

c) Selected demographic group of users. 0 score

d) No description of the sampling strategy. 0 score

2) Sample size:

a) Justified and satisfactory. 1 score

b) Not justified. 0 score

3) Non-Response rate

a) The response rate is satisfactory (≥95%). 1 Score

b) The response rate is unsatisfactory (<95%), or no description. 0 Score

4) Ascertainment of the screening/surveillance tool:

a) Validated screening/surveillance tool. 2 scores

b) Non-validated screening/surveillance tool, but the tool is available or described. 1 score

c) No description of the measurement tool. 0 score

Comparability: (Maximum 1 scores)

1) **The potential confounders were investigated by subgroup analysis or multivariable analysis.**

a) The study investigates potential confounders. 1 score

b) The study does not investigate potential confounders. 0 score

Outcome: (Maximum 3 scores)

1) Assessment of the outcome:

a) Independent blind assessment. 2 scores

b) Record linkage. 2 scores

c) Self report. 1 score

d) No description. 0 score

2) Statistical test:

a) The statistical test used to analyze the data is clearly described and appropriate. 1 score

b) The statistical test is not appropriate, not described or incomplete. 0 score

Table 2; Newcastle-Ottawa Quality Assessment Scale for case control studies used in the systematic review and meta-analysis 2023

	Selection				Comparability	exposure			Total score
Authors	Adequate case definition (1)	Representativeness of the cases (1)	Selection of Controls (1)	Definition of Controls (1)	Comparability of cases and controls on the basis of the design or Analysis for the most important factor or additional factor (2)	Ascertainment of exposure (1)	Same method of ascertainment for cases and controls (1)	Non-Response Rate (1)	
Melkie A et al.(47)	1	1	1	1	2	1	1		9
Sewmehone E et al.(48)	1	1	1	1	2	1	1		9

- The scoring process was made according to Newcastle-Ottawa Quality Assessment Scale adapted for case control studies.

Selection; (maximum 1 star)

1.Is the case definition adequate?

a) yes, with independent validation ♦

b) yes, eg record linkage or based on self-reports

c) no description

2. Representativeness of the cases

a) consecutive or obviously representative series of cases ♦

b) potential for selection biases or not stated

3. Selection of Controls

a) community controls ♦

b) hospital controls

c) no description

4. Definition of Controls

a) no history of disease (endpoint) ♦

b) no description of source

Comparability; (maximum 2 stars)

1. Comparability of cases and controls on the basis of the design or analysis

a) study controls for ____________ (select the most important factor) ♦

b) study controls for any additional factor (These criteria could be modified to indicate specific control for a second important factor.) ♦

Exposure; (maximum 1 stars)

1. Ascertainment of exposure

a) secure record (eg surgical records) ♦

b) structured interview where blind to case/control status ♦

c) interview not blinded to case/control status

d) written self-report or medical record only

e) no description

2. Same method of ascertainment for cases and controls

a) yes ♦

b) no

3. Non-Response Rate

a) same rate for both groups ♦

b) non-respondents described

c) rate different and no designation
